# Maternal and offspring outcomes associated with prescribed ADHD medication in pregnancy: a systematic review

**DOI:** 10.1007/s00737-025-01621-x

**Published:** 2025-10-14

**Authors:** Sarah Tai, Sohum Patel, Kit Downes, Jonathan Rogers, Hannah Chu-Han Huang

**Affiliations:** 1 North London NHS Foundation Trust, London, UK; 2 University College London Hospitals NHS Foundation Trust, London, UK; 3 Division of Psychiatry, University College London, London, UK; 4 National Hospital for Neurology and Neurosurgery, London, UK; 5 South London and Maudsley NHS Foundation Trust, London, UK; 6 Kings College London, Institute of Psychiatry, Child and Adolescent Psychiatry, London, UK

**Keywords:** ADHD, Medication in pregnancy, Perinatal mental health

## Abstract

**Purpose:**

During pregnancy, it is unclear whether women with attention deficit hyperactivity disorder (ADHD) should stop prescribed medication – risking relapse – or continue – risking harm to themselves and their baby. We aimed to conduct a systematic review to examine whether ADHD medications should be continued during pregnancy.

**Methods:**

We searched MEDLINE, Embase, PsycINFO, PubMed, CINAHL, AMED, CENTRAL, Cochrane Library, NHS Knowledge and Library Hub from 1st July 2019 to 1st July 2024, without any restrictions on language, setting, or study type. We supplemented this with relevant studies identified from the references of retrieved studies. Two authors used the Newcastle-Ottawa Scale (NOS) to independently rate the quality of included studies.

**Results:**

Twelve cohort studies were included in the qualitative review. All were deemed high quality (NOS ≥ 7). Seven studies found ADHD medication use during pregnancy had no significant negative effect on maternal or offspring outcomes. One study found continuing ADHD medication reduced the risk of various negative outcomes, and another found stopping ADHD medication may increase the risk of threatened abortion. Three studies concluded that ADHD medication use was associated with negative outcomes: pre-eclampsia, gastroschisis, omphalocele, and transverse limb deficiency. Modafinil was identified as significantly increasing the risk of congenital malformations.

**Conclusion:**

Women taking modafinil should consider stopping it prior to pregnancy. Clinicians should discuss the risks, benefits, and uncertainties of other ADHD medications with women who are pregnant, or considering pregnancy, keeping in mind that the benefits of continuing ADHD medications- where it is effective for an individual- are likely to outweigh the risks.

## Article highlights

- Rates of ADHD in pregnant women are increasing, but research has not kept pace with this.
- There is conflicting evidence as to whether continuing ADHD medication in pregnancy is harmful to mother or baby.
- Over the last 5 years, new evidence has emerged highlighting the benefits of women continuing their ADHD medication during pregnancy, and the risks of stopping.
- There are still risks to continuing ADHD medication in pregnancy, but recent evidence has shown that these risks may be more attributable to ADHD rather than the medication used to treat it.
- Clinicians should discuss the risks and benefits of continuing ADHD medication during pregnancy with women who are pregnant, or considering pregnancy, bearing in mind the negative impact untreated ADHD may have on mother and baby.

## Introduction

Attention Deficit and Hyperactivity Disorder (ADHD) is characterized by a persistent pattern, over at least 6 months, of inattention and/or hyperactivity-impulsivity, which significantly interferes with academic, occupational, or social functioning. Symptoms begin in childhood and can persist into adulthood. (National Institute of Mental Health 2024). These include poor organisational skills, inability to deal with stress, mood swings, and risk-taking behaviour, often with little or no regard for personal safety or the safety of others. Treatment with medications can help relieve symptoms and lessen the impact ADHD has on day-to-day life (National 2021). Left untreated, ADHD can result in a higher risk of substance misuse (Zulauf et al. 2014), depression, anxiety, and suicide (Colvin and Stern 2015; Massuti et al. 2021; Quinn 2014; Stickley et al. 2018). National Institute for Health and Care Excellence (NICE) 2018 Guidelines recommend monotherapy with stimulant medication for adults with ADHD: methylphenidate, lisdexamfetamine or dexamfetamine, and if this fails, non-stimulant medication, such as atomoxetine, or guanfacine, but only under tertiary care supervision (Attention Deficit Hyperactivity Disorder: Diagnosis and Management NICE Guideline, 2018).

As ADHD is classified as a developmental disorder, it precedes and continues throughout pregnancy. As rates of diagnosis of ADHD increase worldwide (McKechnie et al. 2023), rates of ADHD in pregnancy are increasing too (Amikam et al. 2024). With the increased demands of pregnancy and parenting, both patients and clinicians have reported that ADHD symptoms can be more difficult to manage in pregnancy and the post-partum period (Scoten et al. 2024; Young et al. 2020). Pregnant women with ADHD are at higher risk of numerous complications, including, but not limited to, gestational diabetes, pre-eclampsia, and pre-term delivery (Walsh et al. 2022). They are also at higher risk of anxiety and depression in the post-partum period (Andersson et al. 2023). At the same time, treatment with ADHD medication has historically been associated with increased risks of cardiac malformation (Kolding 2021; Koren et al. 2020), pre-eclampsia (Newport et al. 2016; Poulton et al. 2018), pre-term delivery (Cohen et al. 2017), and low birth weight (Philipp 1980). This leaves patients and clinicians with the unenviable task of having to choose between possibly harming the mother and unborn baby by continuing ADHD medication, or possibly harming both by stopping ADHD medication.

Currently, in the UK, NICE guidelines do not cover ADHD medication use in pregnancy. In the USA, the American Journal of Obstetrics and Gynaecology advises that mild to moderate ADHD may be successfully treated in pregnancy with nonpharmacologic treatments, including self-management strategies, but that medication may also be required for moderate to severe ADHD (Scoten et al. 2024). In practice, it is common for care providers to advise patients to stop their ADHD medications if they are contemplating pregnancy or are pregnant (Baker et al. 2022; Young et al. 2020), although practice is changing (Hærvig et al. 2014).

A Danish study published 10 years ago suggested that that the incidence of pregnancies exposed to ADHD medication increased more than 100-fold between 2003 and 2010 (Hærvig et al. 2014). It is likely that it has increased even further since then. Unfortunately, research has not kept pace with this. At time of writing, there were only three systematic reviews looking at ADHD medication use in pregnancy (Jiang 2019; Kittel-Schneider 2021; Li 2020). There were no systematic reviews looking at research conducted over the last 5 years. This is important as recent research has tried to address the issue of confounding by indication, that is, whether outcomes observed were due to mothers having ADHD, rather than being treated for ADHD. There has also been an increase in awareness of the risks of stopping ADHD medication during pregnancy (Russell et al. 2024),with a renewed focus of the impact of a mother’s mental health and wellbeing on maternal and offspring outcomes (Lähdepuro et al. 2023; Mudiyanselage et al. 2024; Satyanarayana et al. 2011).

The aim of this review is to attempt to bridge this gap in knowledge. Specifically, we aimed to ascertain among pregnant women who are on medications for ADHD, the impact on the mother and offspring of continuing those medications compared to stopping them during pregnancy. We concentrate on the last 5 years of research to try and better understand the association between in utero exposure to ADHD medication and a range of maternal and offspring outcomes. We hope that this will add to the evidence base available to clinicians, and help them, and patients, make informed decisions about ADHD medication use in pregnancy.

## Methods

### Search strategy

We conducted our systematic according to the PRISMA (Preferred Reporting Items for Systematic Reviews and Meta-Analysis) statement (Online Resource 3), We searched MEDLINE, Embase, PsycINFO, PubMed, CINAHL, AMED, CENTRAL, Cochrane Library, Google Scholar, NHS Knowledge and Library Hub using a specified search strategy (Online Resource 1) from 1 st July 2019 to 1 st July 2024 (i.e. the last 5 years). We did not impose any restrictions on language or study type. We included grey literature in our search. Two authors (ST and SP) searched references of papers to retrieve additional relevant studies that might have been missed in the electronic search.

### Selection of studies

Two authors (ST and SP) independently selected the studies for this analysis. We included any study of pregnant women of any age, in any setting, receiving the following prescribed ADHD medication (methylphenidate, dexamfetamine, lisdexamfetamine, atomoxetine, guanfacine, clonidine, modafinil) during their pregnancy. We included studies looking at other medications if it was specified that it was for the treatment of ADHD. We excluded studies looking at other medications that were not used for the treatment of ADHD (e.g. antidepressants and antipsychotics). We also excluded studies focused on non-prescription stimulant use. Any disagreements in study selection were resolved by consensus, or, when necessary, by a third author (HH).

### Data extraction

One of two authors (ST and SP) extracted the data from the studies and entered them into a spreadsheet. The following data was extracted: name of first author; publication year; study country; data source; sample size; information on the main exposure, including specific ADHD medications, source of information, exposure to other medications; confounder adjustments; outcomes (including effect measures), and the main conclusion. Any disagreements were resolved by consensus.

### Assessment of study quality

In line with recommendations from the Cochrane Collaboration (Higgins 2024), The authors ST and SP used the Newcastle-Ottawa Scale (NOS)(Wells 2000) to independently rate the quality of the included studies. Any discrepancies were resolved by consensus. The NOS is a validated tool for assessing the quality of observational studies (Stang 2010). It comprises three sections: Selection, Comparability, and Outcome. A study can be awarded a maximum of one star for each numbered item within the Selection and Outcome categories. A maximum of two stars can be given for Comparability. In our review, a study could only receive the maximum of two stars for Comparability if it controlled for (1) ADHD diagnosis/mother being on ADHD medication prior to pregnancy and (2) maternal age, smoking, alcohol, and substance misuse. The higher the score on the NOS, the higher the quality of the study. The maximum score a study can receive is 9 stars. Studies with a score greater than 7 stars are considered to be of high quality. A copy of the adaptation of the NOS we used is included in Online Resource 2.

### Synthesis methods

Due to the anticipated heterogeneity of the study designs and outcomes used, we opted for a narrative synthesis in which studies were grouped according to relevant maternal or offspring outcome. As part of the narrative synthesis of studies, we considered gaps in the literature arising from reporting biases.

## Results

**Fig. 1:**
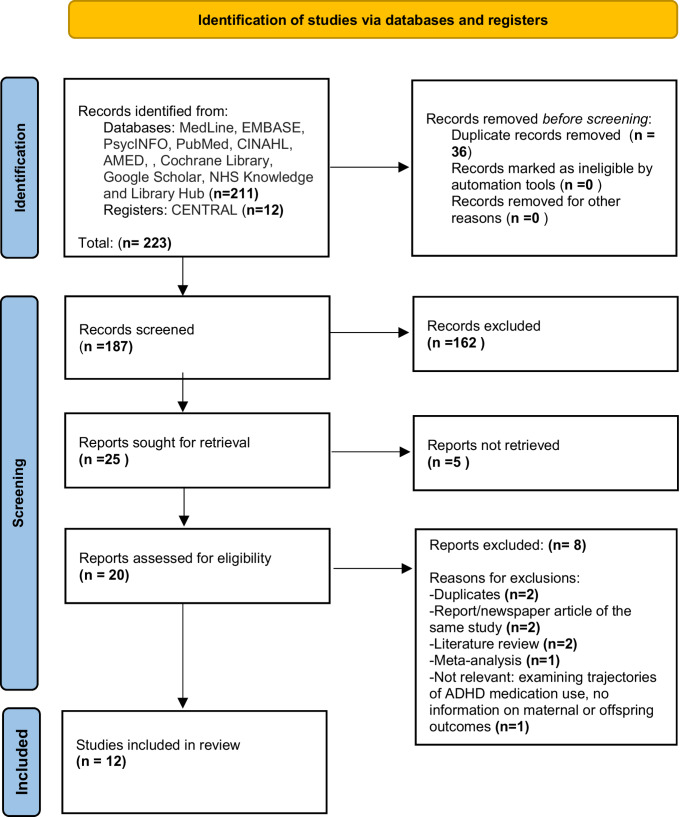
PRISMA (Preferred Reporting Items for Systematic Review and Meta-Analyses) flow diagram for inclusion of the studies examining the association between ADHD medication use during pregnancy and maternal and offspring outcomes

The study selection process is illustrated in Fig. 1. After removal of duplicates, a total of 187 studies and articles were screened independently by authors ST and SP.

The main characteristics of the 12 included studies are shown in Tables 1 and 2. Of the 12 studies, ten sourced data from registry or administrative databases and two sourced data from electronic healthcare records. All 12 were cohort studies, performed in USA (5), Denmark (3), Australia (2), Canada (2), and the Netherlands (1). Sample sizes ranged from 149 to 4,278,460; the total number of included participants was 7,560,673. Nine of the studies defined exposure by prescription records, whilst three defined exposure according to maternal reports at interview.

**Table 1:** Main characteristics of included studies

Citation	Study country	Data source	Sample Size	Exposure	Source of Information
Russell et al. (2024)	Australia	Administrative database linking: 1) The Midwives Notification System (MNS) 2) Monitoring of Drugs of Dependence System (MODDS) 3) Hospital Morbidity Data Collection 4) The WA Registry of Births, Deaths and Marriages.	1688	Dexamfetamine	Prescription and dispensing data from administrative databases
Bang Madsen et al. (2023)	Denmark	Danish Medical Birth Registry	1,092,668	Methylphenidate Amphetamine Dexamfetamine Lisdexamfetamine Modafinil Atomoxetine Clonidine	Prescription and dispensing information from registry data
Lemelin et al. (2021)	Canada	Quebec Pregnancy Child Cohort	166,047	Methylphenidate Amphetamine mixed salts Lisdexamfetamine Dexamfetamine Atomoxetine Guanfacine	1) Data from prescription drug database 2) Maternal Reports
Walsh et al. (2022)	USA	TriNetX database (electronic medical records)	88,653	Amphetamine class Phenidate class Guanfacine Clonidine Bupropion Atomoxetine Viloxazine	Prescription records from TriNetX database
Kolding et al. (2021)	Denmark	1) The Danish foetal medicine database 2) The Danish national patient registry 3) The Danish medical birth registry 4) The Danish Health Services Prescription Database	364,012	Methylphenidate Modafinil Atomoxetine	Prescription and dispensing records from Danish Health Services Prescription Database
Anderson et al. (2020)	USA	The National Birth Defects Prevention Study	43,689	Amphetamine Dextroamfetamine Combination amphetamine/dextroamphetamine Lisdexamfetamine Dexmethylphenidate Methylphenidate Methamphetamine Pemoline Atomoxetine	Maternal interview: computer assisted telephone interview 6 weeks to 24 months after mother’s estimated due date
Suarez et al. (2024)	USA	Health care utilization data from publicly insured (Medicaid) and commercially insured (MarketScan Commercial Claims Database) pregnant individuals	4,278,460	Amphetamine Dextroamfetamine Methylphenidate	Prescription and dispensing records
Szpunar et al. (2023)	USA	Massachusetts General Hospital National Pregnancy Registry for Psychiatric Medications	1988	Mixed amphetamine salts Lisdexamfetamine Methylphenidate Dexmethylphenidate	1) Medical records provided by participants 2) Participant interview: twice during gestation, typically during first and third trimesters, and again at 3 months postpartum
Camacho et al. (2023)	Canada and Australia	Linked health administrative data from: 1) Ontario, Canada (2002-2018) 2) New South Wales, Australia (2003-2012)	693,501	Methylphenidate Amphetamine Dextroamfetamine Lisdexamfetamine	1) Dispensing records from Narcotics Monitoring System (Ontario) 2) Dispensing records from Ontario Drug Benefit Database(Ontario) 3) Dispensing records from database (New South Wales)
Damkier et al. (2020)	Denmark	Danish national health registries	829,656	Modafinil Methylphenidate	Prescription data from registries
Damer et al. (2021)	Netherlands	Electronic patient records of patients within the Isala Psychiatry-Gyanecology-Pediatrics outpatient clinic in Isala hospital	149	Methylphenidate	1) Participant Interview
Rose et al. (2021)	USA	Electronic patient records of patients at the Mayo Clinic Hospital	162	Amphetamine Dextroamfetamine	1) Billing codes 2) Prescription records 3) Both of the above

**Table 2:** Main characteristics of included studies Cont

Citation	Comparison	Co-medicine exposure during pregnancy	Confounder adjustment	Outcomes	Main Results	Newcastle Ottawa Scale Score
Russell et al. (2024)	(1) ADHD + continued DEX during pregnancy (continuers, *n* = 547) (2) ADHD + ceased DEX prior to end of the second trimester or did not take DEX during pregnancy (ceasers, *n* = 297) (3) ADHD + no DEX + no MPH during pregnancy (*n* = 844)	Not measured.	Smoking status, parity, maternal age, hospitalisations for mental health conditions (5 years prior to conception).	Threatened abortion Preeclampsia Hypertension Threatened early labour Premature rupture of membranes Antepartum haemorrhage Precipitate delivery Postpartum haemorrhage Mental health hospitalisation in 3^rd^ Trimester Mental health hospitalisation postpartum (day 0-42) Neonatal special care unit admittance Foetal distress Low birthweight (below 2500 g)	(1)Continuing dexamfetamine during pregnancy is not associated with increase in negative health outcomes for mother or child: pre-eclampsia (OR: 0.64, 95% CI: 0.32-1.26, *p*=0.199), threatened early labour (OR: 1.22, 95% CI: 0.60-2.50, *p*= 0.589), Premature rupture of membranes (OR: 0.55, CI: 0.28-1.10, *p*=0.092), antepartum haemorrhage (OR: 0.82, CI: 0.41-1.64, *p*=0.569), postpartum haemorrhage (OR: 0.83, 95% CI: 0.55-1.26, *p*=0.382), Mental health hospitalisation in 3^rd^ Trimester (OR: 0.70, 95% CI: 0.44-1.10, *p*=0.122), Neonatal special care unit admittance (OR 1.09, 95% CI: 0.82-1.45, *p*=0.560), foetal distress (OR 0.73, 95% CI: 0.49-1.09), *p*= 0.122), low birthweight (OR 0.94, 95% CI: 0.55-1.60, *p*=0.823) : 0.55-1.60, *p*=0.823) (2) Stopping dexamfetamine during pregnancy is associated with increased odds of threatened abortion (OR: 2.28; 95%CI: 1.00, 5.15; *p* = 0.049)	8
Bang Madsen et al. (2023)	(1) Children exposed to ADHD medication during pregnancy (continuers, *n* = 898) (2) Children whose mothers discontinued ADHD medication before pregnancy (ceasers, *n*=1270).	Not measured.	Maternal age at delivery, primiparity, maternal and paternal psychiatric history at delivery, psychiatric in- or outpatient treatment two years prior to pregnancy and until delivery, dispensing of other psychotropic medication during pregnancy, number of hospital visits not related to psychiatry during pregnancy, maternal highest education, calendar year of delivery, maternal self-reported smoking during pregnancy, marital status at delivery.	Neurodevelopmental psychiatric disorders Impairments in vision or hearing Epilepsy Seizures Growth impairment during childhood or adolescence	Continuing ADHD medication in pregnancy is not associated with any increased risk for offspring.(average hazard ratios: 0.97, 95% CI 0.81 to 1.17)	8
Lemelin et al. (2021)	(1) Children exposed to ADHD medication during pregnancy (*n*=133) (2) Children who were not exposed in utero to any ADHD medication (*n*=165 914). Sibling control analysis performed- exposed child matched to unexposed sibling.	Measured in terms of number of medications used: 0, 1-2, 3-5, >6, or not specified. Details of specific medications not provided	Maternal history of ADHD, gestational age, birth weight, newborn gender, maternal age on date of birth, maternal age, urban dweller, welfare recipient, maternal comorbidities in the year before or during pregnancy (diabetes, hypertension, asthma, maternal psychiatric conditions including mood and anxiety disorders, other psychiatric conditions in the year prior to or during pregnancy, maternal lifestyle in the year before pregnancy or during pregnancy (tobacco dependence, alcohol dependence, other drug dependence), health services usage in the year before or during pregnancy (visits to GP, visits to other specialists, ED visit or hospitalization), pregnancy follow up by obstetrician visits, number of medications used other than medication included in study.	ADHD in children: children with at least 1 diagnosis of ADHD according to (a) ICD-10 or (b) having 1 prescription filled for ADHD medications from birth until end of follow up.	(1)In utero exposure to ADHD medication is not associated with an increased risk of ADHD in children. (2)The association between ADHD medication use in pregnancy and increased rates of ADHD diagnosis in children is suggested to be due to genetic and/or family environmental factors. Overall cohort (adjusted hazard ratio= 1.96, 95% CI 1.22–3.15) ADHD pregnant women sub-cohort (adjusted hazard ratio= 1.56; 95% CI 0.93–2.62) Sibling control analysis (adjusted hazard ratio= 1.14; 95% CI 0.62–1.98),	9
Walsh et al. (2022)	(1) Pregnant females with ADHD (*n*=45 737) (2) Pregnant females without ADHD (*n*=42 916) Within pregnant females with ADHD, comparisons made between those on: 1) Stimulant medication 2) Non stimulant medication 3) No medication.	Not measured.	Age, ethnicity, race.	Pre-eclampsia Pre-existing hypertension Gestational hypertension Renal disease Postpartum depression Depressive episode Iron deficiency anaemia Gestational diabetes Eclampsia HPV Pre-term delivery Spontaneous abortion Cardiac disease complicating pregnancy TORCH infections Anaemia complicating pregnancy Malnutrition Early pregnancy haemorrhage Hyperemesis gravidarum.	(1) Mothers with ADHD had higher rates of every negative outcome observed except for HPV infection (2) With the exception of HPV, every outcome studied in the ADHD medication sub-analysis was less common for patients on medications compared to patients without medication use: Pre-eclampsia: OR 0.727, 95% CI 0.639-0.828, p: 0.001 Pre-existing hypertension: OR 0.668, 95% CI 0.565-0.789, p:0.001 Gestational hypertension: OR 0.834, 95% CI 0.751-0.927, p: 0.001 Renal disease: OR 0.525, 95% CI 0.381-0.722, p: 0.001 Postpartum depression: OR 0.809, CI 0.672-0.973, p: 0.024 Depressive episode: OR 0.681, CI 0.611-0.759, p: 0.001 Iron deficiency anaemia: OR 0.742, CI 0.609-0.904, p: 0.003 Gestational diabetes: OR 0.610, CI 0.531-0.7, p: 0.001 Eclampsia: OR 0.665, 95% CI 0.482-0.918, p: 0.012 HPV: OR 1.504, 95% CI 1.065-2.124, p: 0.02 Pre-term delivery: OR: 0.585, 95% CI 0.488-0.701, p: 0.001 Spontaneous abortion: OR 0.638, 95% CI: 0.55-0.74, p: 0.001 Cardiac disease complicating pregnancy: OR 0.544, 95% CI 0.418-0.707, p: 0.001 TORCH infections: OR 0.690, 95% CI: 0.599-0.796, p: 0.001 Anaemia complicating pregnancy: OR 0.621, 95% CI: 0.546-0.705, p: 0.001 Malnutrition: OR 0.491, CI: 0.44-0.549, p: 0.001 Early pregnancy haemorrhage: OR 0.611, 95% CI 0.529-0.706, p: 0.001 Hyperemesis gravidarum: OR 0.606, 95% CI 0.541-0.68), p: 0.001 (3) For approximately half the outcomes, non-stimulant medications had the largest reduction in risk.	8
Kolding et al. (2021)	(1) Pregnancies with redeemed prescriptions for ADHD medication (2) Pregnancies with no redeemed prescriptions for ADHD medication Patients with preconception exposure to ADHD medication (former use) were excluded.	Not measured.	Ethnicity, civil status, parity, age at conception, prepregnant body mass index, smoking during pregnancy, redeemed prescription of a known teratogen (from 2 years preconception), dispensing of other medication (anti hypertensives, antidiabetic agents, antiepileptics, antipsychotics, anxiolytics, hypnotics and sedatives), and maternal hospital diagnoses of ADHD or diabetes 2 years pre-conception date.	(1) Primary outcome: major malformations overall (2) Secondary outcome: malformations of the central nervous system and cardiac malformations Severe cardiac malformations (SCM) as concurrent diagnosis of a cardiac malformation with miscarriage, termination, stillbirth, postnatal death, or cardiac surgery within 1 year of birth	(1) Exposure to methylphenidate was not associated with an increased risk of malformations (prevalence ratio 1.04, 95% CI: 0.70-1.55) (2) There is an increased risk of cardiac malformations (prevalence ratio 1.65, 95% CI: 0.89-3.05, number needed to harm = 92) based on 12 cases among the exposed (3) More data needed on other types of ADHD medication.	7
Anderson et al. (2020)	(1) Mothers exposed to ADHD medication use (*n*= 31 965) (2) Mothers not exposed to ADHD medication use (*n*= 11 724)	Early pregnancy folic acid use.	Age, race/ethnicity, education, income, early pregnancy smoking, early pregnancy alcohol use, early pregnancy folic acid use, pregnancy intention, number of previous births, pre-pregnancy body mass index.	Tetralogy of fallot Coarctation of the aorta Pulmonary valve stenosis Atrial septal defect (secundum or NOS) Neural tube defects Craniosynotosis Cleft palate Cleft lip with or without clef palate Gastroschisis Omphalocele Hypospadias 2/3rd degree Transverse limb deficiency	Statistically significant associations between any early pregnancy ADHD medication use and increased risk for gastroschisis (crude odds ratio cOR: 2.9, 95% CI 1.2-6.9), omphalocele (cOR: 4.0, 95% CI 1.2-13.6), and transverse limb deficiency (cOR 3.3, 95% CI 1.1-9.6).	7
Suarez et al. (2024)	(1) Exposure to amphetamine/dextroamphetamine or methylphenidate during first half (*n*= 25 950) and/or second half of pregnancy (*n* =8188) - continuers (2) Non-exposure to amphetamine/dextroamphetamine or methylphenidate in the second half of pregnancy (*n* = 4 270 272) - ceasers	Antidepressants Anticonvulsants Antipsychotics Anxiolytics/sedatives/hypnotics Benzodiazepines Opioids Corticosteroids Antihypertensives	Maternal age, race and ethnicity, state, delivery year, ADHD, other maternal mental health diagnoses (depression, anxiety, bipolar disorder, other mental health disorders, sleep disorders), proxies for severity of mental health conditions (numbers of psychiatric visits, inpatient and emergency visits for mental health diagnoses, mental health diagnoses, and dispensations of other psychotropic medications), smoking, alcohol, and substance misuse, other medications (antidepressants, anticonvulsants, antipsychotics, anxiolytics/sedatives/hypnotics, benzodiazepines, opioids, corticosteroids, antihypertensives), maternal comorbidities, adequacy of prenatal care, socioeconomic level indicators.	Autism spectrum disorder ADHD and any Neurodevelopmental disorder as a composite of autism spectrum disorder ADHD Specific learning disorders Developmental speech or language disorder Developmental coordination disorder Intellectual disability Behavioural disorder	Amphetamine/dextroamphetamine and methylphenidate exposure in utero are not likely to meaningfully increase the risk of childhood neurodevelopmental disorders. Amphetamine/dextroamphetamine: (autism spectrum disorder: hazard ratio [HR], 0.80; 95% CI, 0.56-1.14]; ADHD: HR, 1.07; 95% CI, 0.89-1.28; any neurodevelopmental disorder: HR, 0.91; 95% CI, 0.81-1.28) Methylphenidate: (autism spectrum disorder: HR, 1.06; 95% CI, 0.62-1.81; any neurodevelopmental disorder: HR, 1.15; 95% CI, 0.97-1.36, ADHD: HR, 1.43; 95% CI, 1.12-1.82, but association did not persist in sensitivity analyses with stricter control for confounding by maternal ADHD)	9
Szpunar et al. (2023)	(1) Women who took a prescription stimulant medication during the first trimester of pregnancy (*n* = 261) (2) Women with psychiatric disorders who did not take any stimulants during pregnancy but who were treated with other psychotropic medications (*n* = 1755)	Prenatal vitamins Selective Serotonin Reuptake Inhibitors (SSRIs) Serotonin-norepinephrine reuptake inhibitors (SNRIs) Tricyclic antidepressants (TCAs) Sedatives/benzodiazepines Other anxiolytics First-generation antipsychotics Second-generation antipsychotics Lithium Anticonvulsants	Age, baseline body mass index, race and ethnicity, college educated, married, first trimester exposure (to cigarettes, alcohol, illicit drugs, prenatal vitamins), first trimester psychotropic use (SSRIs, SNRIs, TCAs, sedatives/benzodiazepines, other anxiolytics, first generation antipsychotics, second general antipsychotics, lithium, anticonvulsants), pregnancy intention, prior pregnancy, primary diagnoses (ADHD, anxiety, bipolar disorder, depression), history of postpartum depression, history of postpartum psychosis.	Presence of a major malformation identified within 6 months of birth Note: major malformations are defined as structural abnormalities with surgical, medical, or cosmetic importance. Chromosomal and single-gene abnormalities and minor abnormalities are excluded from the analysis.	First trimester exposure to stimulant medications: mixed amphetamine salts, lisdexamfetamine, methylphenidate, and dexmethylphenidate do not increase the overall risk of infant major malformations (OR 0.39, 95% CI: 0.09–1.61)	8
Camacho et al. (2023)	(1) Women with at least one dispensing record for methylphenidate, amphetamine, dextroamphetamine or lisdexamfetamine at any time during the first or second trimester (first 180 days from conception date) (2) Women with no dispensing record for either psychostimulants or atomoxetine (a non-stimulant treatment for ADHD) between conception date and date of birth	Buprenorphine Naloxone Methadone Opioids Psychotropics	Age, neighbourhood income quintile, urban residence, calendar year of conception, parity, number of previous admissions, smoking during pregnancy, comorbidities (metabolic conditions, epilepsy, chronic renal disease, thyroid conditions, ADHD, mental and behavioural disorders), psychotropic medications during pregnancy.	Pre-eclampsia Placental abruption Preterm birth (<37 weeks gestation) Low birthweight (<2500 g) Small for gestational age (SGA) Admission to neonatal intensive care unit (NICU)	(1) There are higher rates of adverse perinatal outcomes among pregnancies exposed to psychostimulants: Odds ratios and (95% Confidence Intervals CIs) for Ontario and New South Wales (NSW) Pre-eclampsia: Ontario 2.01 (1.50-2.70), NSW 2.13 (1.18- 3.85) Placental abruption: Ontario 1.88 (1.31-2.70), NSW 1.97 (0.49-7.97) Preterm birth: Ontario 1.80 (1.52-2.13), NSW 1.82 (1.12-2.94) Low birthweight: Ontario 1.73 (1.43-2.09), NSW 2.20 (1.36-3.57) SGA: Ontario 1.15 (0.99-1.34), NSW 0.97 (0.58-1.61) NICU admission: Ontario 2.16 (1.90-2.45), NSW 1.58 (1.09-2.31) but the attenuation of most associations (apart from pre-eclampsia, see below) after adjustment: Placental abruption: Ontario 1.24 (0.79-1.95), Ontario social security 1.61 (0.95-2.71), NSW 1.36 (0.33-5.58) Preterm birth: Ontario 1.25 (1.01-1.55), Ontario social security 1.10 (0.84-1.44), NSW 1.27 (0.77-2.11) Low birthweight: Ontario 1.12 (0.88-1.42), Ontario social security 1.17 (0.88-1.54), NSW 1.44 (0.87-2.36) SGA: Ontario 0.90 (0.76-1.08), Ontario social security 0.98 (0.78-1.24), NSW 0.79 (0.48-1.31) NICU admission: Ontario 1.10 (0.93-1.29), Ontario social security 0.96 (0.78-1.17), NSW 1.10 (0.74-1.62) and likelihood of residual confounding suggests psychostimulant exposure is not a major causal factor for most measured outcomes (2) Findings for pre-eclampsia were inconclusive, exposed pregnancies may benefit from closer monitoring: Weighted Ontario: 2.02 (1.42- 2.88), Weighted Ontario Social security: 1.24 (0.64-2.40) Weighted NSW: 1.50 (0.77-2.94)	8
Damkier et al. (2020)	(1) Pregnancies exposed to modafinil (2) Pregnancies exposed to methylphenidate (3) Pregnancies not exposed to both modafinil nor methylphenidate 1 year prior to and during pregnancy.	Other ATC groups (not nervous system) Nervous system ATC group: NO2 analgesics NO3 antiepileptics NO5 psycholeptics NO6 psychoanaleptics.	Diabetes, hypertension, concomitant use of psychotropic drugs, maternal age, year of delivery, smoking status, BMI.	Major malformations during pregnancy, at delivery, or during the first year of life using the EUROCAT classification of malformations	First trimester in utero exposure to modafinil compared with methylphenidate (OR 3.4, 95% CI 1.2-9.7) or no medication (OR: 2.7, 95% CI 1.1-6.9) is significantly associated with an increased risk of congenital malformations.	7
Damer et al. (2021)	(1) Pregnant women with ADHD diagnosis who continued methylphenidate treatment during pregnancy (continuers) (2) Pregnant Women with ADHD who discontinued methylphenidate treatment during pregnancy (ceasers)	Selective Serotonin Reuptake Inhibitors (SSRIs) Tricyclic Antidepressants (TCAs) Benzodiazepines Antipsychotics	Maternal age, education, prior pregnancy, hypothyroidism, anxiety, psychotic disorder, other developmental disorder, height, weight, BMI, support system, IVF/ICSI, use of other psychotropic drugs (SSRI, TCA, Benzodiazepines, Antipsychotics), alcohol use, smoking, drug use, gestational diabetes, preeclampsia, HELLP syndrome, epidural use, position during delivery, procedure or instrumentation during delivery, meconium, child sequence.	Birth weight 1 min and 5 min Apgar score Congenital malformations (according to 20 week ultrasound).	(1)No significant association between methylphenidate exposure and APGAR score (at 1 min *p*= 0.576, 5 min *p*= 0.281) or neonatal malformations at the 20 week ultrasound scan (*p*= 1.000) (2) Newborn infants exposed to methylphenidate had a higher mean birth weight (*p*=0.049), but after adjustment for covariates this association was not statistically significant (*p*=0.079).	9
Rose et al. (2021)	(1) Pregnant women with ADHD diagnosis or a history of amphetamine-dextroamphetamine use, whose pregnancies were exposed to amphetamine-dextroamphetamine (2) Pregnancies not exposed to amphetamine-dextroamphetamine.	Opioid use Other psychiatric drugs (not specified)	Maternal age, first pregnancy, gravidity, parity, BMI, smoking during pregnancy, history of substance abuse, weight gain during pregnancy, gestational age at delivery, prenatal visits, type of labour, type of delivery, medications (opioid use, psychiatric drugs), infections, migraines, comorbid mental health diagnoses (major depression, anxiety, panic disorder, bipolar disorder), history of substance abuse.	(1) Primary outcome: clinically significant reduction in birthweight (2) Secondary outcome: neonatal complications, including neonatal intensive care unit admissions, neonatal abstinence syndrome, birth defects, and maternal complications (e.g. development of preeclampsia spectrum disorders and post partum haemorrhage).	(1) No differences in infant birthweight (26.9 g, 95%CI: −141–195 g, *p*=0.75), NICU admission (*p*=0.33), or neonatal abstinence syndrome diagnosis (*p*=0.11). (2) No differences observed in maternal pregnancy complications including gestational hypertension or preeclampsia (data not available)	7

The most common medications examined across the 12 studies were stimulants, with methylphenidate being evaluated in 10 studies, and amphetamines in 9. Several studies also looked at atomoxetine (5), guanfacine (2), clonidine (2) and modafinil (2). None of the studies considered exposure duration, nor dosage of ADHD medication.

A range of outcomes were evaluated. For maternal outcomes, these were split into pre-natal (pre-eclampsia, hypertension, gestational diabetes, anaemia, hyperemesis gravidarum, renal disease, depressive episode, human papillomavirus, cardiac disease complicating pregnancy, malnutrition, early pregnancy haemorrhage), birth (placental abruption, post-partum haemorrhage, eclampsia), and post-natal (depression). For offspring outcomes, these were split into pre-natal (threatened abortion, spontaneous abortion, pre-term delivery, small for gestational age), birth (foetal distress, low birthweight) and post-natal (TORCH infections, admission to the neonatal intensive care unit, congenital malformations, cardiac malformations, neurodevelopmental disorders, growth impairment, impairment in vision/hearing, epilepsy, seizures) (Tables 3 and 4).

**Table 3:**
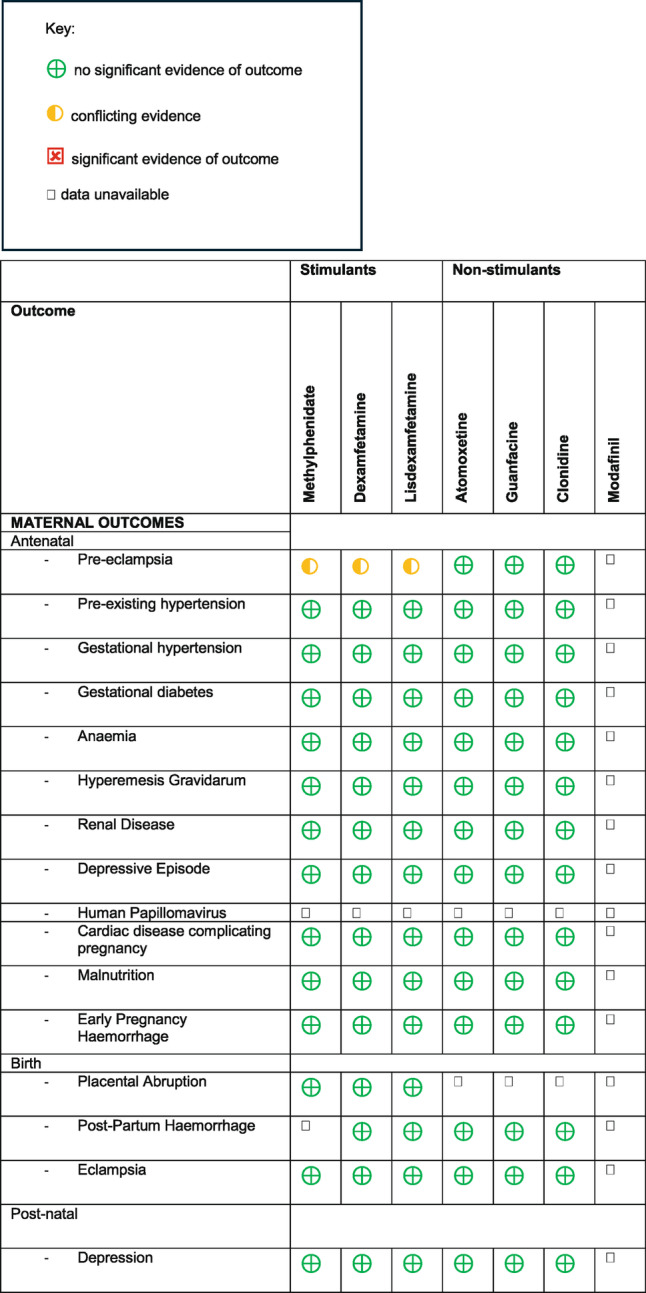
Medication and maternal outcomes

**Table 4:**
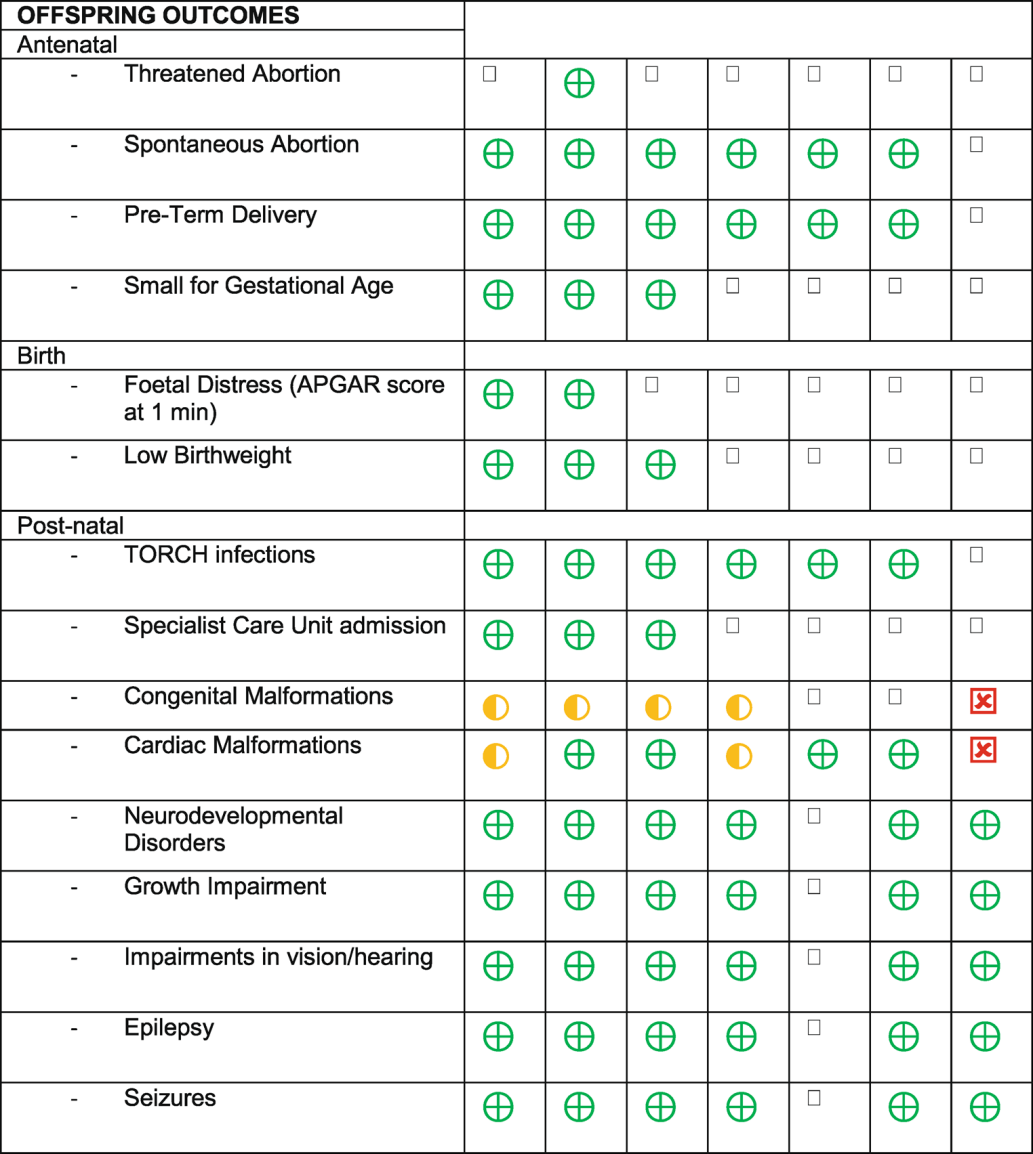
Medication and offspring outcomes

All 12 studies adjusted for a range of confounding factors. These varied between studies, though all controlled for maternal age. Other factors included maternal demographic characteristics (e.g. ethnicity and educational background), maternal health background (e.g. medication use, diagnoses of anxiety, hypothyroidism, epilepsy), and pregnancy characteristics (e.g. parity, gravidity, prenatal visits, type of labour, pregnancy intention).

Half of the studies addressed the issue of confounding by indication. This means the studies took into account the mother’s ADHD diagnosis. Five of the studies (Russell et al. 2024, Bang Madsen et al. 2023; Lemelin et al. 2021; Suarez et al. 2024; Damer et al. 2021) achieved this by comparing outcomes between mothers who continued their prescribed ADHD medications during pregnancy (continuers) and those who ceased taking them (ceasers) In particular, Lemelin et al.(2021) used a sibling analysis which provides better control over confounding by unmeasured family related risk factors shared by siblings (e.g. environmental stress, parenting strategies) that could feasibly be attributed to a maternal ADHD diagnosis. Camacho et al. (2023) included a diagnosis of ADHD on their list of confounders that they adjusted for.

All 12 studies were deemed to be high quality (NOS ≥ 7). Specifically, four studies were given a NOS score of 7, five studies were given a score of 8, and three studies were given a score of 9.

Six studies (Bang Madsen et al. 2024; Lemelin et al. 2021; Suarez et al. 2024; Damer et al. 2021; Szpunar et al., 2023, Rose, Hathcock, White, Borowski & Rivera-Chiauzzi, 2021) found that ADHD medication use during pregnancy did not have a significant negative effect on maternal or offspring outcomes. One study (Kolding et al. 2021) noted that whilst there was no association between methylphenidate and malformations overall in a study with information on both prenatal and postnatal diagnoses, there was an increased risk of cardiac malformations (Prevalence Ratio PR 1.65, 95% CI: 0.89–3.05, number needed to harm = 92) based on 12 cases among the exposed.

Two studies (Walsh, Rosenberg & Hale, 2021, Russell et al. 2024) found a positive effect of ADHD medication use in pregnancy on outcomes. Walsh et al. (2021) found that continuing ADHD medication actually decreased the risk of various negative outcomes (see Table 2 for individual outcome Odds Ratios OR), and Russell et al. (2024) found that stopping prescribed ADHD medication during pregnancy increases the risk of threatened abortion (OR: 2.28; 95%CI: 1.00-5.15; *p* = 0.049). However, it is important to consider that the sample size in Russell et al. (2024) is relatively small (*n* = 1688), and the positive findings are not replicated in larger studies, which may indicate a degree of reporting bias.

Three studies concluded that ADHD medication use during pregnancy was associated with negative perinatal outcomes. Damkier and Broe (2020) noted a significant association between modafinil use in pregnancy and the risk of congenital malformations of the newborn (OR: 2.7, 95% CI 1.1–6.9). Anderson et al. (2020) found statistically significant associations between any early pregnancy ADHD medication use and increased risk for gastroschisis (crude Odds Ratio cOR: 2.9, 95% CI 1.2–6.9), omphalocele (cOR: 4.0, 95% CI 1.2–13.6), and transverse limb deficiency (cOR 3.3, 95% CI 1.1–9.6) Camacho et al. (2023) found higher rates of adverse perinatal outcomes among pregnancies exposed to psychostimulants, including pre-eclampsia, placental abruption, preterm birth (< 37 weeks gestation), low birthweight (< 2500 g), small for gestational age (SGA), and admission to neonatal intensive care unit (NICU). However, the majority of these associations were attenuated after adjustment, and the authors conclude that combined with the likelihood of residual confounding, psychostimulant exposure is likely not a major causal factor for most measured outcomes. The exception is for pre-eclampsia, where odds remained elevated in the weighted analysis of the Ontario cohort (OR 2.02, 95% CI 1.42–2.88), although some attenuation occurred in the New South Wales cohort (weighted OR 1.50, 95% CI 0.77–2.94) and upon restriction to social security beneficiaries (weighted OR 1.24, 95% CI 0.64–2.40), and confidence intervals were wide.

## Discussion

### Main findings

Over the past 5 years, new research has shown that overall, continuing prescribed ADHD medication in pregnancy is not associated with a significant negative effect on maternal or offspring outcomes. In fact, it may have some benefits. This is exciting as whilst previous reviews have all concluded that there is no convincing evidence to indicate that prenatal exposure to ADHD medication results in clinically significant adverse effects, our review is the first to highlight the benefits of continuing, and risks of stopping, ADHD medication during pregnancy. This insight echoes work by Baker et al. (2022) which showed improved mood and family functioning among women who maintain ADHD medication during pregnancy, and will hopefully prompt clinicians to work collaboratively with women and their support networks to balance the risks of perinatal ADHD medication with the risks of inadequately treated ADHD during pregnancy (Scoten et al. 2024).

### Limitations

There are two sets of limitations to be addressed: firstly, Limitations of our review itself, and secondly, Limitations of the evidence we looked at. The most significant limitation of our review is that it only looks at research over the last 5 years. The reason for this was that there have already been three comprehensive systematic reviews (Kittel-Schneider 2021; Li 2020; Jiang 2019) and one meta-analysis (Jiang 2019) of previous research and we did not see any benefit in duplicating this information. A second limitation is that we did not conduct a meta-analysis of the data. The reason for this is that the studies were not sufficiently homogeneous. They looked at a variety of ADHD medications taken at different stages during pregnancy and examined a diverse range of maternal and offspring outcomes. Conducting a meta-analysis on such a clinically diverse set of studies may be meaningless, and genuine differences in effects may be obscured (Higgins 2024). A third limitation is that we did not include any grey literature in our review. Whilst we did find some conference abstracts and news reports in our initial screening, when authors ST and SP contacted the corresponding author to get full texts for review, we did not hear back.

A key limitation of the evidence we looked at is that very few of the studies could confirm whether the prescribed ADHD medication was actually taken. All the studies examined were registry based, and with the exception of Anderson et al. (2020), Szpunar et al. (2023), Damer et al. (2021), Lemelin et al. (2021), relied exclusively on prescription information to determine whether a mother was taking ADHD medication. The four studies mentioned above incorporated a form of interview into their study, but each have their limitations. In Anderson et al. (2020), the interview did not specifically enquire about ADHD and ADHD medication, which the authors posit may result in underreporting of ADHD medication use. There may be recall bias, as ADHD medication use was ascertained via maternal report up to 24 months after the estimated due date, and exposure to ADHD medication was not verified from other sources. Szpunar et al. (2023), and Damer et al. (2021) provide the most convincing evidence of ADHD medication compliance in the form of personalised interviews with the mother specifically enquiring about ADHD medication use, but these are limited by the small sample sizes of exposure to ADHD medication (*n* = 261, *n* = 147 respectively). Lemelin et al. (2021) mentions that data on prescription fillings for ADHD medications were validated against maternal reports but does not describe what this consists of.

A further limitation is the reliance on information from databases. Whilst some of these databases are national registers (e.g. Bang Madsen et al. 2024; Kolding et al., 2021, Damkier & Broe, 2020), most data sources have exclusion criteria which means that the control cohort studied may not be truly representative of the population as a whole. For example, Lemelin et al. (2021) used the Quebec Pregnancy Child Cohort Administrative Database, which only includes women covered by provincial prescription drug insurance. Anderson et al. (2020) uses data from the National Birth Defects Prevention Study (NBDPS), and there may be differences in those participating or not participating in NBDPS. Szpunar et al. (2023), Damer et al. (2021), and Rose et al. (2021) s’ data sources are individual hospitals, and in Damer et al. (2021), a specific psychiatric clinic, which will limit how much the results can be translated to the general population. The use of databases also means that in some studies, there is a reliance on coded diagnoses as proxies for behaviour. For example, in Lemelin et al. (2021), alcohol consumption or smoking was only noted if coded for as alcohol or tobacco dependence. In Suarez et al. (2024), number of psychiatric visits, inpatient admissions and emergency visits were used as a proxy for severity of mental health conditions. This is important as some of these behaviours have an impact on maternal and offspring outcomes, independent to ADHD medication use, or indeed ADHD, and there is a risk that information about these behaviours is missed.

In half the studies reviewed, attempts were made to address confounding by indication. However, in the other half, a pre-existing diagnosis of ADHD or previous treatment with ADHD medication was not addressed. This is significant as the outcomes observed could be due to symptoms of ADHD, rather than the medication used to treat ADHD. None of the studies looked at the nature or severity of maternal ADHD symptoms before or after medication use.

Finally, ethnic representation is an issue; the national registry used in 3 of the 12 studies is Danish, and all the studies reviewed were carried out in developed in countries with primarily Caucasian populations.

### Interpretation

Our review adds to the evidence base from older systematic reviews by Jiang et al. (2019), Li et al. (2020), and Kittel-Schneider et al. (2021), that with the exception of modafinil, continuing ADHD medication in pregnancy is not associated with negative outcomes for mother or child.

The 12 studies we looked at were high quality (NOS > 7), large-scale, registry-based cohort studies, across a range of countries. Half of them addressed confounding by indication, which was highlighted by previous systematic reviews(Kittel-Schneider 2021; Li 2020)^2^ as an area for future research to concentrate on. Five of these studies(Bang Madsen et al. 2023; Damer et al. 2021; Lemelin et al. 2021; Russell et al. 2024; Suarez et al. 2024) went one step further, comparing women who were on ADHD medication prior to pregnancy, who then either continued with this throughout their pregnancy (continuers) or stopped medication during pregnancy (ceasers). These comparisons hold the most clinical value, as the question of whether to continue ADHD medication when a woman finds out she is pregnant is one which clinicians and patients struggle with. Crucially, all five of these studies showed no significant negative maternal or offspring outcomes associated with continuers when compared to those who discontinued.

Nevertheless, it is important to address any negative outcomes. In this review, the main negative outcomes noted were unspecified congenital malformations (Damkier et al. 2020), specific congenital malformations (Anderson et al. 2020), an increased risk of cardiac malformations (Kolding et al. 2021), pre-eclampsia and an increased risk of Neonatal Intensive Care Unit (NICU) admission (Camacho et al. 2023).

Damkier et al. (2020) found that first trimester in utero exposure to modafinil is significantly associated with an increased risk of congenital malformations (OR: 2.7, 95% CI 1.1–6.9). The only other study included in our review that examined modafinil was Kolding et al. (2021), which mainly concentrated on exposure to methylphenidate. In this study, there were relatively small numbers of women exposed to modafinil, and the crude prevalence ratios of major malformations in first-trimester exposed pregnancies (*n* = 30) compared to unexposed (*n* < 5,) was 2.87 (95% confidence interval 1.15–7.15). The authors note that there are too few cases to perform adjusted analyses and conclude that more data is needed. In the same paper, they report that whilst exposure to methylphenidate was not associated with an increased risk of malformations overall (adjusted prevalence ratio aPR 1.04, 95% CI 0.7–1.55), it was associated with an increased risk of cardiac malformations (aPR 1.65, 95% CI 0.89–3.05).

Two other negative outcomes: pre-eclampsia, and an increased risk of NICU admission, were raised by Camacho et al. (2023), and in a previous systematic review and meta-analysis by Jiang et al. (2019), although it is interesting to note both studies reached different conclusions. Camacho et al. (2023) examined nearly 700 000 pregnancies and concluded that whilst stimulant exposure is not a major causal factor for most measured outcomes, there is an uncertainty around a potential association between stimulant medication and pre-eclampsia. Jiang et al.’s (2019) meta-analysis, which pre-dates Camacho et al. (2023), also found an association between ADHD medication use in pregnancy and pre-eclampsia (Risk Ratio RR 1.27; 95% CI 1.11-1.46; *P* < 0.001), but notes that the association disappeared after secondary analysis which took into account confounding associated with maternal ADHD and other psychotropic drug use (RR: 1.13; 95% CI 0.88‐1.45; *P* = 0.363). Instead, across Jiang et al.’s (2019) analysis, an increased risk for NICU admission was seen in infants exposed to ADHD medication (RR 1.88, 95% CI 1.7–2.08). This was also noted in Camacho et al.’s (2023) crude analysis (Ontario: OR 2.16, 95% CI 1.90–2.45, NSW: OR 1.58, 95% CI 1.09–2.31) but there was an attenuation of associations after accounting for measured confounders (Ontario: OR 1.10, 95% CI 0.92–1.29, social security weighted: 0.96, 95% CI 0.78–1.17, NSW: OR 1.10, 95% CI 0.72–1.62).

Finally, Anderson et al. (2020) reported a statistically significant association between any early pregnancy ADHD medication use and an increased risk for gastroschisis (crude odds ratio cOR: 2.9, 95% CI 1.2–6.9), omphalocele (cOR: 4.0, 95% CI 1.2–13.6), and transverse limb deficiency (cOR 3.3, 95% CI 1.1–9.6). However, the authors noted that ADHD medication use among pregnant women in their study was rare, as are the individual birth defects observed to be at increased risk. As such, the absolute risk of a mother having a baby born with each of these specific birth defects after using ADHD medication in pregnancy remains relatively low. This thought was echoed in Kittel-Schneider et al. (2020)’s systematic review, which included the paper by Anderson et al. (2020). Here, the authors felt that these risks are so low that medication should not be stopped if the ADHD is so severe that the treatment is necessary for daily functioning of the affected women.

### Clinical implications

The clinical question at the heart of this review is that if a woman has a diagnosis of ADHD, is on prescribed medication to control her symptoms, and becomes pregnant, should she continue her ADHD medication or stop it?

Based on the evidence to date, with the exception of modafinil, if the ADHD medication prescribed is considered effective, concerns about the impact on a woman’s pregnancy and her offspring should not deter clinicians from continuing to prescribe it, and from women continuing to take it. This is largely in line with the recent guidelines on best practices in managing ADHD in the perinatal period by Scoten et al. (2024), published in the American Journal of Obstetrics and Gynecology, which recommends women with moderate or severe ADHD to consider pharmacotherapy for ADHD.

The caveat is that there are certain high-risk groups (e.g. women who are already at increased risk of pre-eclampsia, or where there is an existing high risk of congenital abnormalities or NICU admission) where the risk versus benefit ratio will need to be carefully considered. These women should have a consultation with a perinatal psychiatrist and obstetrician, and a collaborative approach taken to monitor their pregnancy.

As mentioned above, a notable exception is modafinil. Modafinil, which in the UK is not often used in the treatment of ADHD, should be avoided during pregnancy. If a woman is on it when she realises she is pregnant, she should stop it immediately.

Joint decision-making, where patient preference plays a key role in any decisions made, should be the cornerstone of discussions surrounding any medication use. This is especially true of ADHD in pregnancy. Symptoms of ADHD, benefits and side effects of ADHD medication, and the experience of pregnancy itself, vary widely from woman to woman. In line with Scoten et al.’s (2024) guidelines, it is advisable that all women with diagnosed ADHD, whether or not they are on medication, have an individualised treatment plan. This would involve clinicians working collaboratively with women and their support networks to provide psychoeducation not only about the risks of continuing perinatal ADHD medication during pregnancy, but also about the risks of inadequately treated ADHD during pregnancy. A treatment plan should include psychoeducation, self-management strategies or coaching, psychotherapies, and a discussion around the risks and benefits of continuing ADHD medication. If the clinician feels it is required, a treatment plan could also include increased pre-natal monitoring, with advice around giving birth in a hospital setting where appropriate specialist help is readily available.

Even though the evidence is increasingly pointing towards the benefits of continuing ADHD mediation in pregnancy, should the pregnant woman decide to stop ADHD medication, she should be supported to do so in a safe manner.

This leads us to a trickier question: when in her pregnancy should a pregnant woman stop ADHD medication? The answer to this question is outside the scope of this review, but broadly speaking, it would depend on which risks were felt to be the most pressing, and what is practical. During the first trimester, drugs can produce congenital malformations (Thorpe et al. 2013), and the period of greatest risk is from the third to the eleventh week of pregnancy. During the second and third trimester, drugs can affect the growth or functional development of the foetus, or they can have toxic effects on foetal tissues. Drugs given shortly before term or during labour can have adverse effects on labour or on the neonate after delivery(Joint Formulary Committee 2021). The main negative outcomes mentioned in our review are: specific congenital abnormalities, pre-eclampsia, and an increased risk of NICU admission. By the time a woman discovers she is pregnant, she is usually well into the first trimester, so it may be impossible to completely avoid the first negative outcome (specific congenital abnormalities), unless ADHD medication is stopped prior to conception. The second (pre-eclampsia) and third outcomes (increased risk of NICU admission) could potentially be avoided by stopping ADHD medication as soon as the woman is aware she is pregnant. Crucially, it is important to counsel the pregnant woman that any negative outcome cannot be definitively avoided, as all pregnancies carry a baseline risk, regardless of medication use.

Finally, how should a pregnant woman come off ADHD medication? A sudden cessation of medication can cause biological withdrawal, which may lead to negative outcomes; as shown in Russell et al. (2024), and a worsening of ADHD symptoms. It may be prudent to undertake a gradual reduction in dose over a period of weeks. That being said, the majority of ADHD medications are short-acting and are often only taken on an as-needed basis (e.g. by children during the week when they are at school, with drug “holidays” over the weekend), with no significant evidence of withdrawal (Attention Deficit Hyperactivity Disorder: Withdrawal from Pharmacological Treatment and Drug Holidays NICE Guideline NG87, 2018), so it might be that these risks are overstated.

### Further research

Whilst the weight of evidence seems to support pregnant women with ADHD remaining on ADHD medication during pregnancy, there are several areas that warrant further investigation.

Firstly, the evidence to date has largely relied on registry-based information. Moving forward, this should be coupled with interviews with the mother, and clinicians involved in their care. This would be helpful in confirming medication adherence, the severity of ADHD symptoms, and picking up on alcohol or illicit drug use that may not meet the threshold for diagnostic coding. Secondly, it would be interesting to perform a meta-analysis looking at the negative outcomes raised in this review: pre-eclampsia, congenital abnormalities, specifically: gastroschisis, omphalocele and transverse limb deficiency, and an increased risk of admission to NICU. Thirdly, the studies covered in this review are all based in Western, developed counties. It will be important to look at data from developed Asian countries, and other developing countries, to see if these findings about the safety of ADHD medication in pregnancy can be generalised worldwide.

## Conclusion

Pregnancy is a significant event in a woman’s life. It can be chaotic, stressful, and unpredictable. There are appointments to attend, plans to be made, and often, other children to look after. In a woman with untreated, or undertreated, ADHD, the practical demands, emotional stress, and relationship difficulties can be overwhelming (Baker et al. 2022). Despite this, a recent study found that approximately 60% of women discontinued or interrupted their ADHD medication around pregnancy(Bang Madsen et al. 2024). A common reason given is that women are worried about the negative impact continuing ADHD medication will have on their pregnancy and the health of their baby. This review has found that these worries are, on balance, probably outweighed by the benefits on continuing to take ADHD medications. Moreover, there may be added benefits of continuing ADHD medication in pregnancy. Based on our findings, we have two recommendations. Firstly, we would echo Scoten et al. (2024)’s guidelines in encouraging clinicians to manage the risks and impacts of ADHD in the perinatal period by collaborating with women and their support networks to formulate individualised treatment plans. Secondly, with the exception of modafinil, which should be stopped during pregnancy, we would recommend that if a woman has a diagnosis of ADHD and is on ADHD medication which she finds helpful, she should – after a full discussion of the risks and benefits– consider continuing this throughout pregnancy, with additional monitoring and support from obstetric and perinatal services.

### Registration and protocol

This review is registered on PROSEPRO (CRD4202453893). A review protocol was not registered. A description of amendments to the initial registration is included in Online Resource 4.

## References

[CR1] Amikam U, Badeghiesh A, Baghlaf H, Brown R, Dahan MH (2024) The association between attention deficit hyperactivity disorder and pregnancy, delivery and neonatal outcomes—an evaluation of a population database. BMC Pregnancy Childbirth 24(1):364. 10.1186/s12884-024-06561-5 38750437 10.1186/s12884-024-06561-5 PMC11095018

[CR2] Andersson A, Garcia-Argibay M, Viktorin A, Ghirardi L, Butwicka A, Skoglund C, Madsen B, D’onofrio K, Lichtenstein BM, Tuvblad P, C., Larsson H (2023) Depression and anxiety disorders during the postpartum period in women diagnosed with attention deficit hyperactivity disorder. J Affect Disord 325:817–823. 10.1016/j.jad.2023.01.069 36681302 10.1016/j.jad.2023.01.069

[CR3] Attention deficit hyperactivity (2018) Disorder: diagnosis and management NICE guideline. www.nice.org.uk/guidance/ng87

[CR4] Attention deficit hyperactivity disorder (update) (2018) [I]Withdrawal from Pharmacological treatment and drug holidays NICE guideline NG87 attention deficit hyperactivity disorder (update). FINAL Contents 35192265

[CR5] Baker AS, Wales R, Noe O, Gaccione P, Freeman MP, Cohen LS (2022) The course of ADHD during pregnancy. J Atten Disord 26(2):143–148. 10.1177/1087054720975864 33307923 10.1177/1087054720975864

[CR6] Bang Madsen K, Bliddal M, Skoglund CB, Larsson H, Munk-Olsen T, Madsen MG, Thomsen H, Bergink P, Srinivas V, Cohen C, Brikell JM, Liu X (2024) Attention-deficit hyperactivity disorder (ADHD) medication use trajectories among women in the perinatal period. CNS Drugs 38(4):303–314. 10.1007/s40263-024-01076-1 38489019 10.1007/s40263-024-01076-1 PMC10980654

[CR7] Bang Madsen K, Robakis TK, Liu X, Momen N, Larsson H, Dreier JW, Kildegaard H, Groth JB, Newcorn JH, Thomsen H, Munk-Olsen P, T., Bergink V (2023) In utero exposure to ADHD medication and long-term offspring outcomes. Mol Psychiatry 28(4):1739–1746. 10.1038/s41380-023-01992-6 36759544 10.1038/s41380-023-01992-6

[CR8] Cohen JM, Hernández-Díaz S, Bateman BT, Park Y, Desai RJ, Gray KJ, Patorno E, Mogun H, Huybrechts KF (2017) Placental complications associated with psychostimulant use in pregnancy. Obstet Gynecol 130(6):1192–1201. 10.1097/AOG.0000000000002362 29112657 10.1097/AOG.0000000000002362 PMC5709205

[CR9] Colvin MK, Stern TA (2015) Diagnosis, evaluation, and treatment of attention-deficit/hyperactivity disorder. J Clin Psychiatry 76(09):e1148–e1148. 10.4088/JCP.12040vr1c 26455686 10.4088/JCP.12040vr1c

[CR10] Damer EA, Edens MA, van der Loos MLM, van Esenkbrink J, Bunkers I, van Roon EN, ter Horst PGJ (2021) Fifteen years’ experience with methylphenidate for attention-deficit disorder during pregnancy: effects on birth weight, Apgar score and congenital malformation rates. Gen Hosp Psychiatry 73:9–15. 10.1016/j.genhosppsych.2021.09.003 34507078 10.1016/j.genhosppsych.2021.09.003

[CR11] Hærvig KB, Mortensen LH, Hansen AV, Strandberg-Larsen K (2014) Use of ADHD medication during pregnancy from 1999 to 2010: a Danish register‐based study. Pharmacoepidemiol Drug Saf 23(5):526–533. 10.1002/pds.3600 24590619 10.1002/pds.3600

[CR12] Higgins J, Thomas J, Chandler J, Cumpston M, Li T, Page M, Welch V (eds) (2024), August Cochrane Handbook for Systematic Reviews of Interventions version 6.5. Cochrane 10.1002/14651858.ED000142 PMC10284251 31643080

[CR13] Jiang, H. Yin, Zhang, X., Jiang, C. Ming, Fu H. Bin (2019) Maternal and neonatal outcomes after exposure to ADHD medication during pregnancy: A systematic review and meta-analysis. Pharmacoepidemiology and drug safety, vol 28. John Wiley and Sons Ltd, pp 288–295. 3 10.1002/pds.4716 10.1002/pds.4716 30585374

[CR14] Joint Formulary Committee (2021) British National formulary (online). BMJ and Pharmaceutical, London

[CR15] Kittel-Schneider S, Quednow BB, Leutritz AL, McNeill RV, Reif A (2021) Parental ADHD in pregnancy and the postpartum period – A systematic review. Neuroscience and biobehavioral reviews, vol 124. Elsevier Ltd, pp 63–77. 10.1016/j.neubiorev.2021.01.002 10.1016/j.neubiorev.2021.01.002 33516734

[CR16] Kolding L, Ehrenstein V, Pedersen L, Sandager P, Petersen OB, Uldbjerg N, Pedersen LH (2021) Associations between ADHD medication use in pregnancy and severe malformations based on prenatal and postnatal diagnoses. J Clin Psychiatry 82(1). 10.4088/JCP.20m13458 10.4088/JCP.20m13458 33406323

[CR17] Koren G, Barer Y, Ornoy A (2020) Fetal safety of methylphenidate—a scoping review and meta analysis. Reprod Toxicol 93:230–234. 10.1016/j.reprotox.2020.03.003 32169555 10.1016/j.reprotox.2020.03.003

[CR18] Lähdepuro A, Lahti-Pulkkinen M, Pyhälä R, Tuovinen S, Lahti J, Heinonen K, Laivuori H, Villa PM, Reynolds RM, Kajantie E, Girchenko P, Räikkönen K (2023) Positive maternal mental health during pregnancy and mental and behavioral disorders in children: a prospective pregnancy cohort study. J Child Psychol Psychiatry 64(5):807–816. 10.1111/jcpp.13625 35524467 10.1111/jcpp.13625

[CR19] Lemelin M, Sheehy O, Zhao JP, Bérard A (2021) Maternal ADHD medication use during pregnancy and the risk of ADHD in children: importance of genetic predispositions and impact of using a sibling analysis. Eur Neuropsychopharmacol 44:66–78. 10.1016/j.euroneuro.2021.01.003 33461830 10.1016/j.euroneuro.2021.01.003

[CR20] Li L, Sujan AC, Butwicka A, Chang Z, Cortese S, Quinn P, Viktorin A, Öberg AS, D’Onofrio BM, Larsson H (2020) Associations of Prescribed ADHD Medication in Pregnancy with Pregnancy-Related and Offspring Outcomes: A Systematic Review. In CNS Drugs (Vol. 34, Issue 7, pp. 731–747). Adis. 10.1007/s40263-020-00728-2 10.1007/s40263-020-00728-2 PMC7338246 32333292

[CR21] Massuti R, Moreira-Maia CR, Campani F, Sônego M, Amaro J, Akutagava-Martins GC, Tessari L, Polanczyk GV, Cortese S, Rohde LA (2021) Assessing undertreatment and overtreatment/misuse of ADHD medications in children and adolescents across continents: a systematic review and meta-analysis. Neurosci Biobehav Rev 128:64–73. 10.1016/j.neubiorev.2021.06.001 34089763 10.1016/j.neubiorev.2021.06.001

[CR22] McKechnie DGJ, O’Nions E, Dunsmuir S, Petersen I (2023) Attention-deficit hyperactivity disorder diagnoses and prescriptions in UK primary care, 2000–2018: population-based cohort study. BJPsych Open 9(4):e121. 10.1192/bjo.2023.512 37455585 10.1192/bjo.2023.512 PMC10375867

[CR23] Mudiyanselage SB, Arachchige Dona W, Angeles S, Majmudar MR, Marembo I, Tan M, Price EJ, Watts A, Gold JJ, Abimanyi-Ochom J (2024) The impact of maternal health on child’s health outcomes during the first five years of child’s life in countries with health systems similar to Australia: a systematic review. PLoS ONE 19(3):e0295295. 10.1371/journal.pone.0295295 38457392 10.1371/journal.pone.0295295 PMC10923423

[CR24] National Health Service (2021) Attention deficit hyperactivity disorder (ADHD): symptoms and treatment. National Health Service Online

[CR25] National Institute of Mental Health (2024) Attention deficit hyperactivity disorder. U.S, Department of Health and Human Services, National Institutes of Health

[CR26] Newport DJ, Hostetter AL, Juul SH, Porterfield SM, Knight BT, Stowe ZN (2016) Prenatal psychostimulant and antidepressant exposure and risk of hypertensive disorders of pregnancy. J Clin Psychiatry 77(11):1538–1545. 10.4088/JCP.15m10506 28076672 10.4088/JCP.15m10506

[CR27] Philipp E (1980) Guanfacine in the treatment of hypertension due to pre-eclamptic toxaemia in Thirty women. Br J Clin Pharmacol 10(1). 10.1111/j.1365-2125.1980.tb04921.x 10.1111/j.1365-2125.1980.tb04921.x PMC1430123 6994768

[CR28] Poulton AS, Armstrong B, Nanan RK (2018) Perinatal outcomes of women diagnosed with Attention-Deficit/Hyperactivity disorder: an Australian population-based cohort study. CNS Drugs 32(4):377–386. 10.1007/s40263-018-0505-9 29557079 10.1007/s40263-018-0505-9

[CR29] Quinn PO, Madhoo M (2014) A Review of Attention-Deficit/Hyperactivity Disorder in Women and Girls. The Primary Care Companion For CNS Disorders. 10.4088/PCC.13r01596 25317366 10.4088/PCC.13r01596 PMC4195638

[CR30] Russell DJ, Wyrwoll CS, Preen DB, Kelty E (2024) Investigating maternal and neonatal health outcomes associated with continuing or ceasing dexamphetamine treatment for women with attention-deficit hyperactivity disorder during pregnancy: a retrospective cohort study. Arch Womens Ment Health 27(5):785–794. 10.1007/s00737-024-01450-4 38424254 10.1007/s00737-024-01450-4 PMC11405422

[CR31] Satyanarayana V, Lukose A, Srinivasan K (2011) Maternal mental health in pregnancy and child behavior. Indian J Psychiatry 53(4):351. 10.4103/0019-5545.91911 22303046 10.4103/0019-5545.91911 PMC3267349

[CR32] Scoten O, Tabi K, Paquette V, Carrion P, Ryan D, Radonjic NV, Whitham EA, Hippman C (2024) Attention-deficit/hyperactivity disorder in pregnancy and the postpartum period. Am J Obstet Gynecol 231(1):19–35. 10.1016/j.ajog.2024.02.297 38432409 10.1016/j.ajog.2024.02.297

[CR33] Stang A (2010) Critical evaluation of the Newcastle-ottawa scale for the assessment of the quality of nonrandomized studies in meta-analyses. Eur J Epidemiol 25(9):603–605. 10.1007/s10654-010-9491-z 20652370 10.1007/s10654-010-9491-z

[CR34] Stickley A, Tachimori H, Inoue Y, Shinkai T, Yoshimura R, Nakamura J, Morita G, Nishii S, Tokutsu Y, Otsuka Y, Egashira K, Inoue M, Kubo T, Tesen H, Takashima N, Tominaga H, Koyanagi A, Kamio Y (2018) Attention-deficit/hyperactivity disorder symptoms and suicidal behavior in adult psychiatric outpatients. J Neuropsychiatry Clin Neurosci 72(9):713–722. 10.1111/pcn.12685 10.1111/pcn.12685 29845681

[CR35] Suarez EA, Bateman BT, Hernandez-Diaz S, Straub L, McDougle CJ, Wisner KL, Gray KJ, Pennell PB, Lester B, Zhu Y, Mogun H, Huybrechts KF (2024) Prescription stimulant use during pregnancy and risk of neurodevelopmental disorders in children. JAMA Psychiatr 81(5):477–488. 10.1001/jamapsychiatry.2023.5073 10.1001/jamapsychiatry.2023.5073 PMC10809143 38265792

[CR36] Thorpe PG, Gilboa SM, Hernandez-Diaz S, Lind J, Cragan JD, Briggs G, Kweder S, Friedman JM, Mitchell AA, Honein MA, National Birth Defects Prevention Study (2013) Medications in the first trimester of pregnancy: most common exposures and critical gaps in understanding fetal risk. Pharmacoepidemiol Drug Saf 22(9):1013–1018. 10.1002/pds.3495 23893932 10.1002/pds.3495 PMC3996804

[CR37] Walsh CJ, Rosenberg SL, Hale EW (2022) Obstetric complications in mothers with ADHD. Front Reprod Health. 10.3389/frph.2022.1040824 36419963 10.3389/frph.2022.1040824 PMC9678343

[CR38] Wells G, Shea B, O’Connell D, Peterson J, Welch V, Losos M, Tugwell P (2000) The Newcastle-Ottawa Scale (NOS) for assessing the quality if nonrandomized studies in meta-analyses

[CR39] Young S, Adamo N, Ásgeirsdóttir BB, Branney P, Beckett M, Colley W, Cubbin S, Deeley Q, Farrag E, Gudjonsson G, Hill P, Hollingdale J, Kilic O, Lloyd T, Mason P, Paliokosta E, Perecherla S, Sedgwick J, Skirrow C, Woodhouse E (2020) Females with ADHD: an expert consensus statement taking a lifespan approach providing guidance for the identification and treatment of attention-deficit/ hyperactivity disorder in girls and women. BMC Psychiatry 20(1):404. 10.1186/s12888-020-02707-9 32787804 10.1186/s12888-020-02707-9 PMC7422602

[CR40] Zulauf CA, Sprich SE, Safren SA, Wilens TE (2014) The complicated relationship between attention deficit/hyperactivity disorder and substance use disorders. Curr Psychiatry Rep 16(3):436. 10.1007/s11920-013-0436-6 24526271 10.1007/s11920-013-0436-6 PMC4414493

[CR41] Anderson, K. N., Dutton, A. C., Broussard, C. S., Farr, S. L., Lind, J. N., Visser, S. N., Ailes, E. C., Shapira, S. K., Reefhuis, J., & Tinker, S. C. (2020). ADHD Medication Use During Pregnancy and Risk for Selected Birth Defects: National Birth Defects Prevention Study, 1998-2011. Journal of Attention Disorders, 24(3), 479–489. 10.1177/1087054718759753 10.1177/1087054718759753 PMC6119527 29519207

[CR42] Camacho X, Zoega H, Gomes T, Schaffer AL, Henry D, Pearson SA, Vigod S, Havard A (2023) The association between psychostimulant use in pregnancy and adverse maternal and neonatal outcomes: Results from a distributed analysis in two similar jurisdictions. International Journal of Epidemiology 52(1):190–202. 10.1093/ije/dyac180 36135973 10.1093/ije/dyac180 PMC9908060

[CR43] Damkier, P., & Broe, A. (2020). First-Trimester Pregnancy Exposure to Modafinil and Risk of Congenital Malformations. JAMA, 323(4), 374. 10.1001/jama.2019.20008 10.1001/jama.2019.20008 PMC6990936 31990303

[CR44] Liberati A, Altman DG, Tetzlaff J, Mulrow C, Gøtzsche PC, Ioannidis JPA, Clarke M, Devereaux PJ, Kleijnen J, Moher D (2009) The PRISMA Statement for Reporting Systematic Reviews and Meta-Analyses of Studies That Evaluate Health Care Interventions: Explanation and Elaboration. PLoS Medicine 6(7). 10.1371/journal.pmed.1000100 10.1371/journal.pmed.1000100 PMC2707010 19621070

[CR45] Rose SJ, Hathcock MA, White WM, Borowski K, Rivera-Chiauzzi EY (2021) Amphetamine-Dextroamphetamine and Pregnancy: Neonatal Outcomes After Prenatal Prescription Mixed Amphetamine Exposure. Journal of Attention Disorders 25(9):1295–1301. 10.1177/1087054719896857 31931669 10.1177/1087054719896857

[CR46] Szpunar, M. J., Freeman, M. P., Kobylski, L. A., Rossa, E. T., Gaccione, P., Chitayat, D., Viguera, A. C., & Cohen, L. S. (2023). Risk of Major Malformations in Infants After First-Trimester Exposure to Stimulants: Results From the Massachusetts General Hospital National Pregnancy Registry for Psychiatric Medications. Journal of Clinical Psychopharmacology, 43(4), 326–332. 10.1097/JCP.0000000000001702 10.1097/JCP.0000000000001702 37235505

